# Metabolic and oxidative stress markers in Wistar rats after 2 months on a high-fat diet

**DOI:** 10.1186/1758-5996-6-130

**Published:** 2014-11-28

**Authors:** Nathalie Auberval, Stéphanie Dal, William Bietiger, Michel Pinget, Nathalie Jeandidier, Elisa Maillard-Pedracini, Valérie Schini-Kerth, Séverine Sigrist

**Affiliations:** UMR DIATHEC, EA 7294, Centre Européen d’Etude du Diabète, Université de Strasbourg, Fédération de Médecine Translationnelle de Strasbourg, Bld René Leriche, 67200 Strasbourg, France; Structure d’Endocrinologie, Diabète –Nutrition et Addictologie, Pôle NUDE, Hôpitaux Universitaires de Strasbourg, (HUS), 67000 Strasbourg, France; Département de Pharmacologie et Physicochimie, UMR 7213 Centre National de la Recherche Scientifique, Université de Strasbourg, Faculté de Pharmacie, BP60024, 67401 Illkirch, France

**Keywords:** Metabolic syndrome, High-fat diet, Oxidative stress, Complications

## Abstract

**Background:**

Metabolic syndrome is associated with an increased risk of cardiovascular and hepatic complications. Oxidative stress in metabolic tissues has emerged as a universal feature of metabolic syndrome and its co-morbidities. We aimed to develop a rapidly and easily induced model of metabolic syndrome in rats to evaluate its impact on plasma and tissue oxidative stress.

**Materials and methods:**

Metabolic syndrome was induced in rats using a high-fat diet (HFD), and these rats were compared to rats fed a normal diet (ND) for 2 months. Metabolic control was determined by measuring body weight, blood glucose, triglycerides, lipid peroxidation and protein carbonylation in plasma. Insulinemia was evaluated through the measure of C-peptide. Histological analysis was performed on the pancreas, liver and blood vessels.

**Results:**

After 2 months, the HFD induced an increase in body weight, insulin and triglycerides. Liver steatosis was also observed in the HFD group, which was associated with an increase in glycogen storage. In the pancreas, the HFD induced islet hyperplasia. Tissue oxidative stress was also increased in the liver, pancreas and blood vessels, but plasma oxidative stress remained unchanged.

**Conclusion:**

This paper reports the development of a fast and easy model of rat metabolic syndrome associated with tissue oxidative stress. This model may be a good tool for the biological validation of drugs or antioxidants to limit or prevent the complications of metabolic syndrome.

## Background

The consumption of diets high in energy (protein, fat and carbohydrates) has become a habit in modern societies despite the reduction in daily energetic needs from 3000 to 2200 kcal [1]. These preferences for high-calorie or high-fat diets (HFDs) lead to an obesogenic environment for humans, increasing the risk of developing diseases associated with metabolic disorders such as obesity and metabolic syndrome (MS). MS has become a public health problem throughout the world and is defined by a combination of several metabolic abnormalities: impaired glucose tolerance or insulin resistance, impaired blood pressure, dyslipidemia (increase in triglycerides and high-density lipoprotein cholesterol), cardiovascular alterations and central obesity [2, 3]. These metabolic disorders have been identified as risk factors for the development of severe complications. In obese patients, ectopic storage of fat occurs in particular organs and tissues that are not normally involved in fat storage, such as cardiovascular tissues including blood vessels [4]. Moreover, fat-engorged adipocytes and fat-laden myocytes are resistant to the signalling effects of insulin, leading to the storage of lipids and triglycerides in several tissues, including the pancreas and liver [5]. The presence of saturated fat in the liver in turn causes insulin resistance in this organ [6, 7], leading to NAFLDs (non-alcoholic fatty liver diseases), particularly steatosis [8]. Other metabolic alterations may also be added to the list of known and identified factors leading to MS, with oxidative stress (OS) as one candidate factor [9]. Close links exist between MS and OS [10–12] due to an imbalance between pro-oxidant and anti-oxidant species in favour of oxidised entities [13]. Indeed, patients with metabolic syndrome exhibit damage due to OS [14]. Insulin resistance and inflammation increase reactive oxygen species (ROS) production and OS [15] through superoxide anion production and the decreased levels of antioxidative enzymes such as superoxide dismutase and glutathione peroxidase [16]. This leads to end products of lipid peroxidation such as malondialdehyde (MDA) and carbonylated proteins [17] in the plasma and to the accumulation of ROS in blood vessels [18]. Moreover, the accumulation of insulin in MS stimulates lipogenesis and further hepatic glycogen synthesis [19, 20].

Faced with the widespread morbidity induced by MS, scientists are trying to understand the impact of environmental factors on humans using *in vivo* models. Many animal models of MS have been developed, including spontaneous transgenic models and diet-induced models. The most common spontaneous or transgenic MS models are i) ob/ob mice (autosomal recessive disease) [21, 22]; ii) obese “yellow mice” (autosomal dominant disease) [23]; iii) NZO (New Zealand Obese) obese mice (polygenic disease) [24], and iv) obese Zucker rats [25]. Dietary models combining high-calorie diets with reductions in physical activity are easier and cheaper and reflect the natural history of metabolic syndrome. For example, in the spiny mouse (*Acomys cahirinus*), a rich sucrose diet induces glucose intolerance and hyperinsulinemia without hyperglycemia or obesity, while a high-lipid diet induces obesity and hyperglycemia [26]. The sand rat (*Psammomys obesus*) develops obesity and hyperinsulinism associated with glucose intolerance after three months on a high-calorie diet. Moreover, after six months, diet can induce insulin-dependence [27]. HFD is well-known to induce some metabolic disorders, and the consequences of HFD are completely dependent on the composition and duration of the diet [28, 29]. Therefore, these data suggest that it is possible to develop a model of MS by changing the composition of the diet. However, it is important to take the age of the rats in these models into account. The age of Wistar rats appears to be related to the development of metabolic syndrome [3]. Few studies have characterised the metabolic alterations and their consequences during a short-term diet. Therefore, the dual effects of age and the duration of the diet could create a bias in these models.

The aim of this study was to develop a rapid and easy model of MS and to characterise the metabolic and tissue alterations in that model by subtracting age-related effects. MS was induced by feeding young and healthy Wistar rats a HFD containing high levels of lipids. The animals were then followed for two months to understand the impact of HFD over a short period on the appearance of MS and its consequences for metabolic parameters and tissue alterations.

## Materials and methods

### Animals

This study was performed in accordance with the “Guide for the Care and Use of Laboratory Animals” published by the US National Institutes of Health (NIH publication No. 85–23, revised 1996). Every effort was made to minimise animal suffering and to reduce the numbers of animals used. The laboratory has been licensed by the Department of Veterinary Service (license N° B67-482-28). The study protocol has been approved through the license of S. Sigrist (N°67-318).

Male Wistar rats (DEPRE, Saint Doulchard, France) 6 weeks of age were housed in a climate-controlled room (22 ± 2°C and 60% relative humidity) with a 12 h light/dark cycle. The rats had *ad libitum* access to water and food.

The animals were randomly divided into two equal groups. The ND group (Normal Diet, n = 6) received the standard diet (A04, SAFE, Augy, France) with an Atwater fuel energy of 2.8 kcal/g (Table 1). The HFD group (High-Fat Diet, n = 10) received the WESTERN RD diet (SDS, Special Diets Services, Saint Gratien, France) for 2 months, which has an Atwater fuel energy of 4.6 kcal/g (Table 1).

**Table 1:** **Composition of ND and HFD**

	A04 (ND)	WESTERN RD (HFD)
**Crude fat (%)**	3.1	21.4
**Crude protein (%)**	16.1	17.5
**Crude fibre (%)**	3.9	3.5
**Ash (%)**	5.1	4.1
**Carbohydrate (%)**	59.9	50
**Moisture (%)**	11.9	3.5
**Atwater fuel energy**	**2.8 kcal/g**	**4.6 kcal/g**

### Metabolic follow-up

All rats were weighed once a week throughout the study period. Before sacrifice, the rats were fasted overnight, and rat tail blood samples were collected into Microtainer™ tubes containing lithium heparin (Becton Dickinson, Franklin Lakes, NJ, USA) during fasting and 30 minutes after re-feeding. These samples were then stored at -80°C. Glycemia was measured using the glucose RTU® kit (Biomérieux, Craponne, France) and expressed in g/L. The measure of C-peptide was preferred rather than the measure of insulin to evaluate insulinemia using a rat C-peptide ELISA kit (Mercodia, Uppsala, Sweden) and expressed in pmol/L.

Glucose Tolerance Test was performed after an overnight fast. A 2 g/kg glucose 20% solution was injected intraperitoneally (IpGTT). Plasma glucose concentration was evaluated using a glucometer at baseline and 15, 30, 45, 60, 90, and 120 minutes after the glucose load (AccuCheck Go; Roche Diagnostics, Meylan, France). Areas under the curve (AUC) were determined to compare groups.

Insulin resistance was evaluated by calculating the homeostasis model assessment (HOMA2): HOMA2 was calculated from fasting plasma glucose and fasting serum C-peptide using the HOMA2 model calculator (http://www.dtu.ox.ac.uk/homa).

### Animal sacrifice and collection of the biological material

By the end of the experimental period, all of the rats were sacrificed. After anaesthesia induced by a mixture of xylazine (Rompun® 2%, Bayer, Puteaux, France) and ketamine (Imalgène® 1000, Merial, Lyon, France) at a dose of 100 μL/100 g, blood was drawn from the abdominal aorta into tubes containing EDTA. Plasma samples were then stored at -80°C. A piece of fresh liver was weighed and placed in a tube containing sodium acetate buffer for glycogen determination.

Additional liver and pancreas samples were either immersed in 4% paraformaldehyde for subsequent paraffin inclusion or embedded in Tissue-Tek® OCT (Electron Microscopy Sciences, Hatfield, PA, USA) and directly frozen in liquid nitrogen and stored at -80°C. A piece of the mesenteric artery was also obtained and embedded in OCT.

### Lipid peroxidation

The lipid peroxidation protocol was adapted from the AMDCC protocol (Animal Models of Diabetic Complications Consortium). Briefly, 100 μL of plasma was mixed with 200 μL of 10% trichloroacetic acid (TCA, Fisher Scientific), cooled on ice for 15 minutes, and then centrifuged at 2200 × g for 15 minutes at 4°C. The supernatants were then mixed with 200 μL of TBA, 0.67% (Sigma) and heated in a dry bath at 95°C for 1 hour. Then, 150 μL of each solution was transferred into a 96-well plate, and the absorbance was measured at 550 nm. Lipid peroxidation (μmol/L) was reported with respect to the total amount of protein in the sample as measured by the Bradford method [30].

### Protein carbonylation

Protein carbonylation was determined by the OxiSelect™ assay (Cell Biolabs, Inc., San Diego, CA, USA). This protocol was performed according to the manufacturer’s instructions, with plasma samples prepared at a final concentration of 10 mg/mL of total protein. The quantity of carbonylated proteins was measured by spectrophotometry at 450 nm (iMark, Bio-Rad) and expressed as nmol/mg of total protein, as determined by the Bradford assay [30].

### Triglycerides content

Triglycerides were determined using the Triglycerides Quantification Kit (BioVision Research Products, Mountain View, CA, USA) according to the manufacturer’s instructions. Samples were measured at 550 nm and concentrations were expressed in mmol/L.

### Total cholesterol content

Enzymatic determination of total cholesterol was performed using the Cholesterol RTU™ (Biomérieux, Marcy-l’Etoile, France) according to the manufacturer’s instructions. Samples were measured at 550 nm and concentrations were expressed in mmol/L.

### Hepatic glycogen content

Fresh liver tissue (100 mg) was mixed with a high-speed homogeniser (Polytron PT MR2100, Kinematica AG, Luzern, Switzerland) in a sodium acetate buffer (0.2 M, pH 4.5). An aliquot of homogenate was mixed with amyloglucosidase (AMGD, Roche Diagnostic) and incubated at 55°C for 90 min to degrade the glycogen into glucose residuals. An aliquot without AMGD was used as a free glucose control. Each aliquot was centrifuged (10 min, 4000 × g, 4°C), and the supernatants were retained for glucose determination. The samples (10 μL) or reference samples of glucose (0–1 g/L) were incubated with a glucose detection reagent (ortho-dianisidine + glucose oxidase/peroxidase reagent) at room temperature for 10 min. The amount of glucose was determined by measuring the absorbance at 450 nm. Samples were analysed in duplicate and the results were determined as μg glycogen per μg used liver (adapted from [31]).

### Histology of liver and pancreas tissue

Paraformaldehyde-fixed liver and pancreas specimens were embedded in paraffin blocks and cut into 4 μm thick sections. The sections were then stained with Masson trichrome (MT) according to standard procedures. The islet surface distribution was determined in the pancreas. At least three sections were examined per animal. The islet surface area was measured using Nikon NIS Elements Br software (Nikon, Tokyo, Japan), and the distributions were reported using box and whiskers plots with median, first and third quartile and extreme values. In the liver, the degree of steatosis was defined on sections according to Kleiner *et al.*[32], with a maximum score of 3. No accumulation of lipids occurs in the pancreas.

### Study of tissue oxidative stress

The oxidative fluorescent dye dihydroethidine (DHE, Sigma-Aldrich) was used to evaluate *in situ* formation of ROS by a method described by Dal-Ros *et al.*[33]. Unfixed liver, pancreas and mesenteric artery tissues were cut into 10 μm thick sections, treated with DHE (2.5 μM), and incubated in a light-protected humidified chamber at 37°C for 30 min. The level of ROS was determined using a microscope, and whole tissue fluorescence was quantified with NIS-Elements BR (Nikon). The fluorescence intensity of tissues was quantified in five arbitrarily selected fields, and the mean value for each section was calculated. The results were expressed by comparing the fluorescence between the control ND and HFD groups.

### Statistical analysis

Statistical analysis was performed using STATISTICA® version 10, Statsoft. Differences between the two groups (ND, HFD) were evaluated with t-tests after validating normality. The data are reported as the mean ± SEM for all parameters except for the islet surface distribution, where the data were reported as median (interquartile range). Statistically significant influences of diet are denoted by (*). P values <0.05 were considered statistically significant.

## Results

### Effect of HFD on body weight and the metabolic response

**Figure 1:**
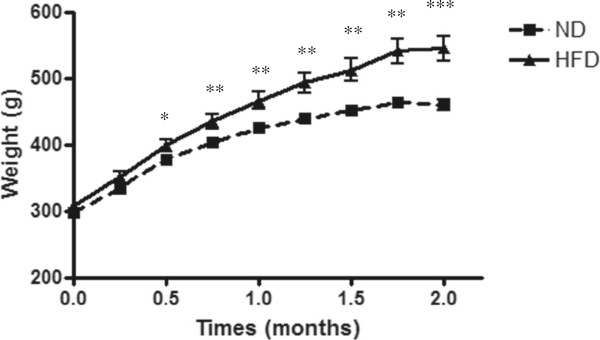
**Body weight of ND (■) or HFD (**▲**) rats over 2 months.** The data are expressed as the mean ± SEM with several animals per group (ND, n = 6 and HFD, n = 10). *: significant difference between the body weight of ND and HFD rats (**: p < 0.01 and ***: p < 0.001).

**Figure 2:**
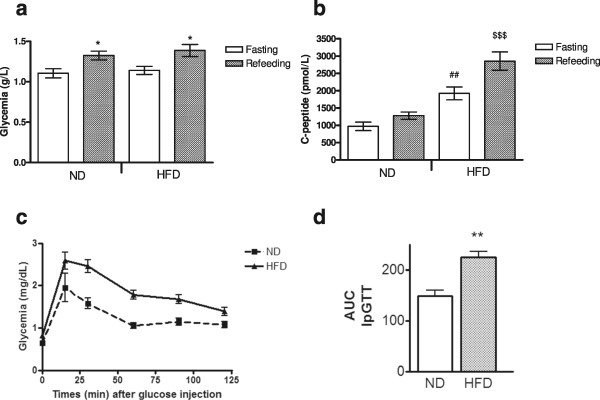
**Effect of 2 months of ND or HFD on glucose metabolism of rats.** Glyceamia **(A)** and c-peptidemia **(B)** under fasting and refeeding conditions, glucose tolerance test (IpGTT) **(C)**, and IpGTT area under the curve **(D)** The data are expressed as the mean ± SEM (ND: n = 6 and HFD: n = 10). *: p < 0.05: significant difference between fasting and refeeding conditions for glycemia or C-peptidemia between ND and HFD rats. ##: p < 0.01: significant difference between ND and HFD C-peptidemia under fasting conditions. $$$ p < 0.001: significant difference between ND and HFD C-peptidemia under refeeding conditions.

HFD induced a significant increase in body weight (Figure 1) compared to ND rats (p < 0.01) after only 1 month on this diet. This difference increased after 2 months (HFD: 536.8 ± 11.9 g; ND: 459.3 ± 8.1 g; p < 0.001). After 2 months, fasting glycemia (Figure 2A) was comparable between ND and HFD rats. Moreover, 30 minutes after refeeding, glycemia was significantly increased (p < 0.05) compared to fasting conditions but comparable between the ND and HFD groups. However, HFD induced a significant increase in C-peptide levels after fasting (p < 0.01) or 30 minutes after re-feeding (p < 0.001). In fact, the HOMA2 was significantly increased in HFD rats compared to ND (p < 0.001). HOMA2 of the HFD group was higher than 2.4 confirming insulin resistance (Table 2). Finally, glucose tolerance testing showed that.

**Table 2:** **Effect of ND or HFD on plasma parameters after 2 months**

	ND	HFD
**Lipid peroxides TBARS** **(μmol/L/mg of total proteins)**	2.04 ± 0.29	3.07 ± 0.60
**Carbonylated proteins** **(nmol/mg of total proteins)**	0.25 ± 0.03	0.32 ± 0.03
**Triglycerides** **(mmol/L)**	1.58 ± 0.07	8.46 ± 1.19**
**Cholesterol** **(mmol/L)**	2.92 ± 0.41	3.43 ± 0.47
**HOMA2**	2,4 ± 0,12	4,5 ± 0,4***

The data are expressed as the mean ± SEM (ND, n = 6 and HFD, n = 10. **: p < 0.01; *** p < 0.001: significant differences between ND and HFD rats).

HFD increased glucose intolerance of rats (Figure 2C) (AUC IpGTT: 224.41 ± 12.01 *vs.* 148.41 ± 10.84 mg/dL per minute, p < 0.01, Figure 2D).

The levels of lipid peroxidation and protein carbonylation were unchanged between the ND and HFD groups after 2 months. The same results were obtained for cholesterol. However, HFD induced a significant increase in triglyceride levels (8.46 ± 1.19 mmol/L *vs.* 1.58 ± 0.07 mmol/L, p < 0.01) (Table 2).

### Effect of the HFD on the pancreas and islets

**Figure 3:**
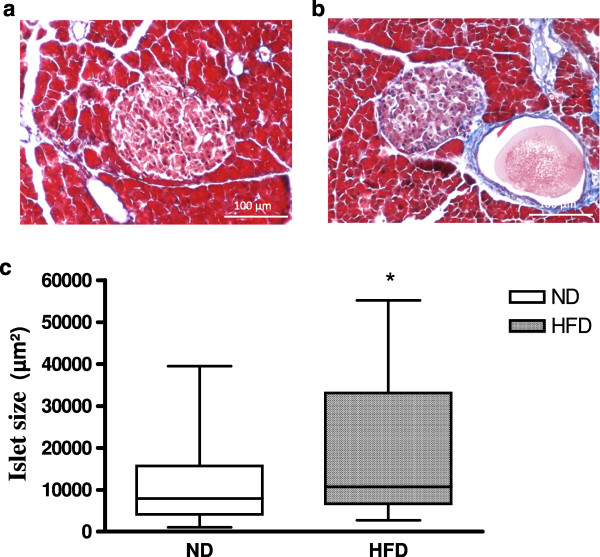
**Effect of ND (A) or HFD (B) on rat pancreatic islets after 2 months.** Islets were stained with Masson’s trichrome, and the surface area was determined **(C)**. *: significant difference in islet surface area between ND and HFD rats (*: p < 0.05).

Histological analysis of the pancreas demonstrated a preservation of the pancreas structure without any fibrosis. Indeed, islets were ovoid in form, with cytoplasms stained pink or light purple and nuclei stained dark purple (Figure 3A and 3B). Moreover, significant staining of islets with insulin was observed, reflecting functional islets (data not shown). However, islets from HFD rats were bigger than those from ND rats, and some holes were observed in pancreatic tissue that were not observed in ND rats. HDF induced a significant increase in islet surface are (p < 0.05), (Figure 3C).

### Effect of HFD on liver structure and glycogen content

**Figure 4:**
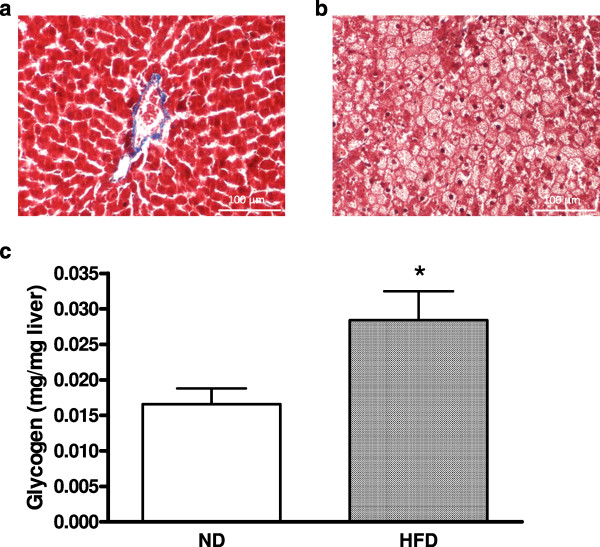
**Effect of ND or HFD on the rat liver after 2 months.** Tissues were stained with Masson’s trichrome **(A and B)**, and glycogen was extracted from the liver and quantified **(C)**. The data are expressed as the mean ± SEM with five animals per group. *: significant difference in hepatic glycogen between the ND and HFD groups (*: p < 0.05).

Histological analysis of hepatic tissue from ND rats showed that the cytoplasm of hepatocytes were homogeneously coloured red, the nuclei were well-defined by violet staining, and cells were organised radially around centro-lobular veins without fibrosis, resulting in a steatosis score of 0, according to Kleiner *et al.*[32] (Figure 4A). However, widespread steatosis was observed in tissues from HFD rats. Indeed, hepatocytes were ballooned and contained many vacuoles with droplets (Figure 4B). Nuclei were less visible and were at the edge of the cytoplasm, suggesting a level of steatosis greater than 70% (steatosis score = 3; Kleiner *et al.*[34]). Moreover, HFD also induced a significant increase in hepatic glycogen content in the liver compared to ND rats (p < 0.05) (Figure 4C).

### Effect of HFD on ROS production in the pancreas, liver and mesenteric artery

**Figure 5:**
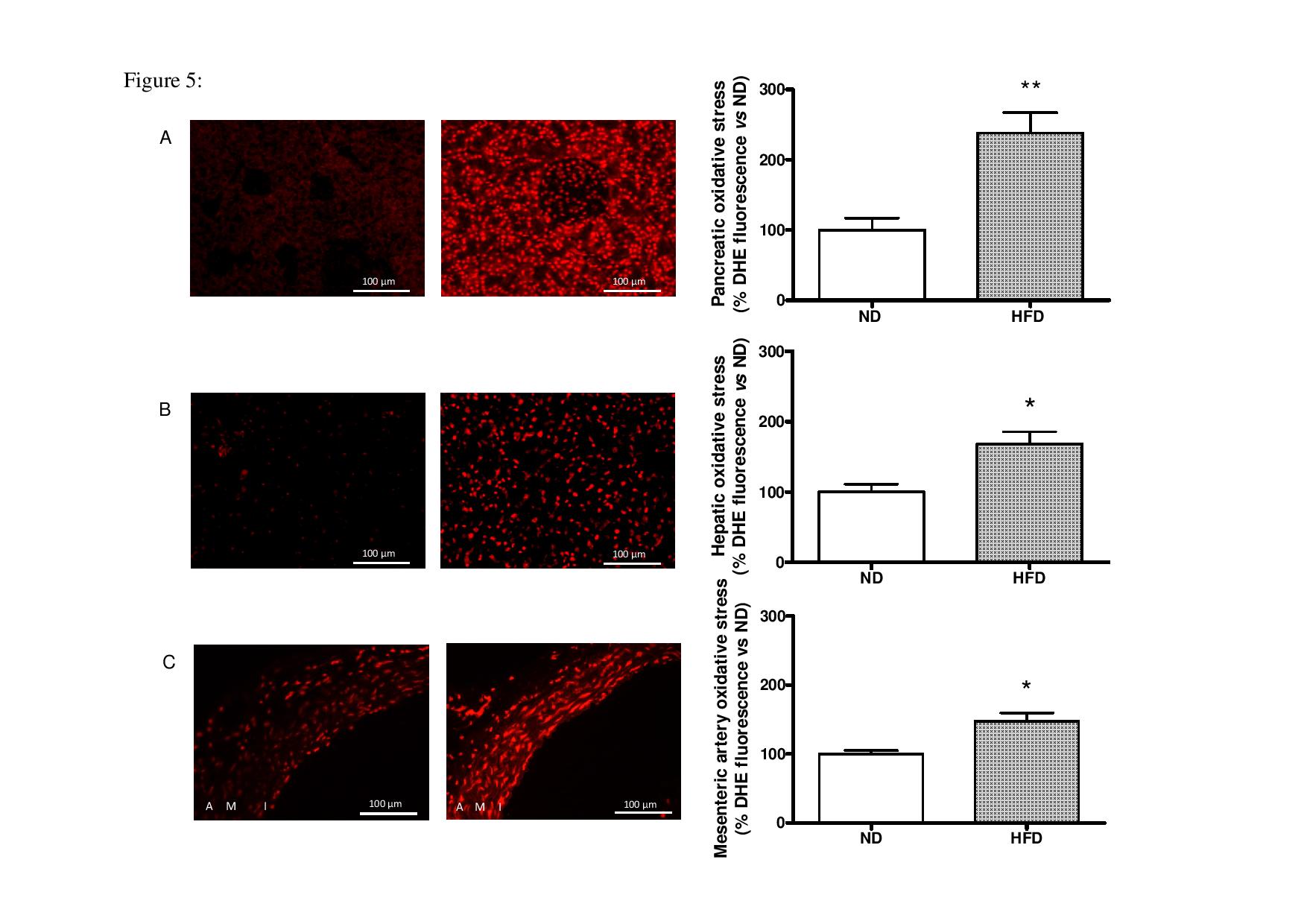
**Effects of ND or HFD on oxidative stress in the pancreas (A), liver (B) and mesenteric artery (C) after 2 months.** Tissues from ND (left pictures) or HFD (right pictures) were stained with DHE, and the amount of DHE was quantified for several animals per group (ND, n = 4-5-4 and HFD, n = 4-4-5, respectively). *: significant difference in oxidative stress between the ND and HFD groups (*: p < 0.05 and **: p < 0.01).

HFD induced a significant increase in ROS production in the pancreas, liver and the mesenteric artery after two months compared to the ND (Figure 5). The level of oxidative stress was increased to 238.2 ± 28.6% (p < 0.01) in the pancreas, 168.2 ± 17.3% (p < 0.05) in the liver and 147.9 ± 11.6% (p < 0.05) in the mesenteric artery.

## Discussion

In this study, we developed a rapidly and easily induced model of MS by feeding healthy Wistar rats with a HFD. After only 2 months, this special diet containing seven fold more fat (21%) and almost the same level of carbohydrates (50%) as the common diet (3% and 60%, respectively), provoked an obesogenic environment which in turn caused several metabolic disorders: obesity, insulin resistance, hypertriglyceridemia and widespread alterations in the tissues studied here, including hyperplasia of pancreatic islets and hepatic steatosis. These data correspond with the accepted definition of MS. Moreover, these characteristics were associated with OS in these tissues and in blood vessels, but not in plasma as assessed by lipid peroxidation and protein carbonylation.

The metabolic follow-up showed that short-term HFD induced a significant weight gain in rats after one month, which was confirmed after 2 months. This weight gain correlates well to the literature, where many studies report weight gains in HFD rats treated for different periods [34–39]. In parallel with this weight gain, HFD rats exhibit increases in abdominal fat mass (data not shown), indicating the development of obesity in these rats. This obesity is accompanied by a significant increase in C-peptide levels in fasting and re-feeding conditions without changes in glycemia but associated with an insulin resistance confirmed by a significant increase of HOMA2. The same profiles were reported in Wistar rats fed for 8 weeks with high-fat, low-carbohydrate diets [39]. Other studies reported both hyperglycemia and hyperinsulinemia after only 7 weeks of a HFD containing higher fat than in our study (30-35%) [35]. This suggests that after 8 weeks of HFD with only 21% fat, we were able to induce hyperinsulinism without any change in glycemia. These results also suggest that to induce hyperglycemia, we should increase the level of fat in our diet to 35%. Furthermore, a diet rich in fat (40%) but poor in carbohydrates (14%) was not sufficient to increase glycemia after 2 months [39]. In fact, short-term MS can be induced by a diet containing at least 20% fat combined with approximately 50% carbohydrates.

In our study, hyperinsulinemia was associated with hypertriglyceridemia, which was characterised by high plasma triglyceride levels in HFD rats. The same profile was also observed by Sasidharan *et al.*[40] with a diet containing a comparable fat level. Conversely, the HFD induced a dramatic increase in hepatic glycogen storage reflecting the activation of glycogenesis, characteristic of MS. In contrast, de Castro *et al.*[41] required more time (13 weeks) to observe an increase in glycogen deposition in the liver using a diet with a high level of fat (40%) and a lower level of carbohydrates (21%). Surprisingly, Gauthier and colleagues [42] reported a decrease in hepatic glycogen storage in rats fed with a diet with the same level of carbohydrates (42%) and a higher level of fat (36%) without requiring the same long time period.

Short term HFD also induced important alterations in pancreatic and hepatic tissues and in blood vessels. In the pancreas, islet hyperplasia was observed in HFD rats in response to hyperinsulinism, with observations including enlarged cells, holes and an increase in the islet surface area. The same trend was also reported in mice fed with a diet with a higher level of fat (60%) for 8, 10 and 12 weeks [43–45] and in rats [46] fed a higher level of fat (60%) for 10 weeks. In the liver, the hepatocytes of HFD rats exhibit small and widespread vacuoles in the cytoplasm which are smaller than the nuclei of hepatic cells. This suggests the presence of microvesicular lipid deposition characterising hepatic steatosis [32]. This accumulation of lipids only occurs at hepatic levels. Vacuoles were also observed in the livers of rats fed with HFD with higher levels of fat (58% and 45%) for nearly the same period of time [34, 47].

HFD rats also exhibited an increase in the quantities of superoxide anions in pancreatic and hepatic tissue and the mesenteric artery as revealed by dihydroethidium (DHE) staining compared to rats fed with the ND. This indicates the development of OS combined with MS, as previously reported [10–12]. However, OS was only present in tissues, as we reported no effects of short-term HFD plasma OS (protein carbonylation and lipid peroxidation). A longer period of treatment with HFD (more than 2 months) led to the detection of plasma OS due to increases in the levels of oxidised proteins and lipids (data not shown). Some studies reported the appearance of lipid peroxidation in the plasma after 8 weeks [48] or 10 weeks [49] of HFD containing more fat (32 and 36%) than in our study. In contrast, few or no studies have reported the analysis of carbonylated plasma proteins in HFD model rats. Only Cole and colleagues [50] showed no effect of high-fat (55%), low-carbohydrate (16%) diets on mitochondrial proteins after 3 weeks.

The model developed here is essentially linked to the hypercaloric diet with high fat (21%) and high carbohydrate contents (50%), without age-effects. Ghezzi *et al.*[3] demonstrated that aging causes the spontaneous development of obesity and insulin resistance in adult and mature rats fed with ND. Other studies have also reported aging-related endothelial dysfunction associated with oxidative stress [33]. Moreover, most MS models result in complications that are often irreversible. In contrast, our model leads to reversible tissue alterations (data not shown). In fact, we have shown in our laboratory that the stop of the HFD feeding or the introduction of antioxidant in the diet could reduce tissue oxidative stress, restore normal insulin and decrease lipid accumulation in the liver what makes this model an ideal tool for the development of particular diet or the validation of biological effectiveness of antioxidant compounds.

This study demonstrates that our rapid and simple model of MS induction by feeding young Wistar rats with a HFD is a true model of MS as reported in humans. The model was associated with metabolic disorders, reversible damage, tissue alterations and OS in the pancreas, liver and blood vessels, without age-linked effects. This model presents many advantages, making it a good tool for studies of MS prevention, including studies assessing new therapeutic strategies such as antioxidative molecules.

## Abbreviations

AMDCC: Animal models of diabetic complications consortium
AMGD: Amyloglucosidase
DHE: Dihydroethidium
EDTA: Ethylene diaminetetraacetic acid
ELISA: Enzyme-linked immunosorbent assay
HFD: High fat diet
HOMA: Homeostasis Model Assessment
MDA: Malondialdehyde
MS: Metabolic syndrome
MT: Masson’s trichrome
NAFLDs: Non-alcoholic fatty liver disease
ND: Normal diet
NZO: New Zealand obese
OCT: Optimal cutting temperature
OS: Oxidative stress
ROS: Reactive oxygen species
SDS: Special diet services
SEM: Standard error of the mean
TBA: Thiobarbituric acid
TCA: Trichloroacetic acid.
